# Profile of microRNA in Giant Panda Blood: A Resource for Immune-Related and Novel microRNAs

**DOI:** 10.1371/journal.pone.0143242

**Published:** 2015-11-23

**Authors:** Mingyu Yang, Lianming Du, Wujiao Li, Fujun Shen, Zhenxin Fan, Zuoyi Jian, Rong Hou, Yongmei Shen, Bisong Yue, Xiuyue Zhang

**Affiliations:** 1 Key Laboratory of Bio-resources and Eco-environment, Ministry of Education, College of Life Science, Sichuan University, Chengdu, Sichuan, 610064, P.R. China; 2 The Key Laboratory for Conservation Biology of Endangered Wildlife, Sichuan Province, Chengdu Research Base of Giant Panda Breeding, Chengdu, Sichuan, 610081, China; 3 Gooddoctor pharmaceutical Group, NO.88 Yingkou Road, Chengdu, Sichuan, 610000, China; Kunming University of Science and Technology, CHINA

## Abstract

The giant panda (*Ailuropoda melanoleuca*) is one of the world’s most beloved endangered mammals. Although the draft genome of this species had been assembled, little was known about the composition of its microRNAs (miRNAs) or their functional profiles. Recent studies demonstrated that changes in the expression of miRNAs are associated with immunity. In this study, miRNAs were extracted from the blood of four healthy giant pandas and sequenced by Illumina next generation sequencing technology. As determined by miRNA screening, a total of 276 conserved miRNAs and 51 novel putative miRNAs candidates were detected. After differential expression analysis, we noticed that the expressions of 7 miRNAs were significantly up-regulated in young giant pandas compared with that of adults. Moreover, 2 miRNAs were up-regulated in female giant pandas and 1 in the male individuals. Target gene prediction suggested that the miRNAs of giant panda might be relevant to the expressions of 4,602 downstream genes. Subseuqently, the predicted target genes were conducted to KEGG (Kyoto Encyclopedia of Genes and Genomes) pathway analysis and we found that these genes were mainly involved in host immunity, including the Ras signaling pathway, the PI3K-Akt signaling pathway, and the MAPK signaling pathway. In conclusion, our results provide the first miRNA profiles of giant panda blood, and the predicted functional analyses may open an avenue for further study of giant panda immunity.

## Introduction

MicroRNAs (miRNAs), as small non-coding (~22nt) RNAs, are key regulators of gene expression [1,2]. Precursor miRNA would form the mature functional miRNA after its stems loop structure is cut by two RNase III enzymes, Drosha and Dicer. The mature miRNAs are incorporated into the RNA-induced silencing complex (RISC), which binds to the 3’UTR protein-coding transcripts and represses the transcript translation or degrades the mRNA in mammals [3]. This leads many biological processes to be regulated, including development, reproduction, apoptosis, proliferation, pathogenesis, and lipometabolism [4–6]. It means that the abnormal expression of miRNAs may cause many diseases such as cardiac disease and cancer [7]. For example, the miR-150 was down-regulated in serum of arterial fibrillation patients, while the expression of miR-1, miR-134, miR-186, miR-208, miR-233, and miR-499 increased in serum of acute myocardial infarction patients [8–10]. Meanwhile, many diseases are related to immunity regulation, and several miRNAs have been shown to be important in immune functions [11,12]. For example, MiR-146 is implicated in numerous cancers and inflammatory diseases and has been confirmed to regulate inflammatory responses in several different cell types [13,14]. Thus, miRNAs represent an important target for potential therapeutic and diagnostic agents [15].

The giant panda, *Ailuropoda melanoleuca*, is an endangered mammal, which was once widespread in southern China but is now found only in Western China in Sichuan, Shanxi, and Gansu provinces of China [16, 17]. This species has received widespread attention and its draft genome has been assembled [17]. Although many miRNAs have been identified in mammals, there have been not yet been any reports about miRNAs of the giant panda [18, 19]. Blood, as a relatively easy tissue to isolate, is the main part of the immune system, and many diseases can be discovered through blood [14]. Therefore, it is more suitable and convenient to use blood to identify miRNAs related to immune and diseases. In this study, we sequenced the small RNA of four giant pandas blood through the next generation sequencing (NGS) technology. We aim to identify the giant panda’s conserved and novel miRNAs and to investigate the effect of miRNAs in giant panda blood on immunity.

## Materials and Methods

### Ethics Statement

The blood samples were collected in routine physical examination through a veterinarian of Chengdu Research Base of the Giant Panda Breeding who has years of experience to take care of giant pandas. All samples collection and utility protocols were approved by the Chengdu Institute of Biology Animal Use Ethics who responsible for Chengdu Research Base of the Giant Panda Breeding. Our experimental procedures complied with the current laws on animal welfare and research in China.

### Sample Preparation

The blood samples were collected from four healthy giant pandas which were living in the Chengdu Research Base of the Giant Panda Breeding in Chengdu City, Sichuan Province, China. With the help of a veterinarian, bloods were drawn from the left forearms of panda in a routine examination. The fresh blood samples were collected in Paxgene RNA Blood Tube and immediately stored at -80°C until use. The four Giant Pandas contained two young individuals (age 5 of male and 6 of female, sub-adult) and two adult individuals (age 12 of male and 18 of female), because age 5 and 6 belong to sub-adult and age 12 and 18 belong to adult [20,21]. (Table 1)

**Table 1:** Summary the gender and age for all samples.

sample	M05	F06	M12	F18
age	5	6	12	18
sex	male	female	male	female

### Small RNA library construction and sequencing

Total RNAs were extracted from four samples of these bloods using Trizol reagent (Invitrogen, Carlsbad, CA, USA) in accordance with the manufacturer’s protocol. The integrity and quality of total RNAs were checked by the 2100 Bioanalyzer (Agilent Technologies). For every sample, 10μg of total RNAs with Sample Prep Kit (Illumina, USA) was used for constructing the library according to the manufacturer’s instructions. Briefly, five steps were followed to construct the libraries. 1) Small RNAs, which enrich about 15–35 nt molecules, were excised from total RNAs through 15% Tris-Borate-EDTA (TBE) denaturing polyacrylamide gel electrophoresis (PAGE). 2) RNA adaptors 3’ and 5’ were ligated to total RNAs with T4 RNA ligase. 3) The adaptor-ligated sRNAs were used as the templates for cDNA synthesis. 4) The cDNA were amplified using PCR with appropriate cycles. 5) After separation of the target DNA fragment with PAGE gel electrophoresis, gel was extracted to construct the sequencing libraries. And then small RNAs were sequenced with the proprietary Solexa sequencing-by-synthesis method through the Illumina Genome Analyzer (SanDiego, CA, USA). Genomics Institute (Novogene and BGI, China) carry out the sequencing.

### Data processing

After sequencing the reads, four steps needed to be followed to remove reads that are not considered appropriate for further analysis. First, the low-quality reads which the value of the quality of alkali base less than 5 accounted for 50% of the entire reads should to be removed. Second, remove reads with 5' primer contaminants or without 3' primer insert fragments. Third, after trimming the 3’adptor, remove the reads including poly A, poly T, poly C, and poly G. Fourth, remove reads less than 18nt. The remaining sequences, called clean reads, were retained for further analysis.

In order to prevent non-miRNAs (rRNA, tRNA, snRNA, snoRNA, etc) sequences from disturbing the analyses, the clean reads were compared against the non-miRNA in database (Genbank, Repeat sequence, Rfam) using BLASTN to remove and annotate non-miRNAs. During annotation, some sRNAs could be assigned to more than one category; therefore, to ensure that every sRNA was given only one annotation, we established a following rule: rRNA > tRNA > scRNA > snRNA > snoRNA> repeat > exon > intron. The remaining reads were analyzed using miRDeep2 software [22]. The MiRDeep2 mapper program using the bowtie algorithm was used to map the remaining reads on the whole giant panda genome with less than 1 base mismatch [23]. The MiRBase database provides both mature miRNA and miRNA precursors for the miRDeep2 program to identify the conserved miRNAs and predict novel miRNAs [24]. The criteria of these conserved and predicted novel miRNAs corresponded to a score of reads above 5 points, a true positive prediction average percentage greater than 90%, and a signal-to-noise ratio larger than 21 estimated by miRDeep2. According to the sequence of miRNA precursor, the miRNAs which belong to the same precursor were considered the same kind of miRNAs. Then, miRDeep2 calculates the expression of miRNAs based on the number of miRNAs in different categories. We predicted the secondary structure of all conserved miRNAs and novel miRNAs trough miRDeep2 randfold program.

### Differential expression of miRNAs

To find the differential expression of miRNAs, we compare all samples (adult and young giant pandas; male and female giant pandas), using Log2-ratio, respectively. First of all, the expression of miRNA in the four samples needs to be normalized to get the expression of transcripts per million.

Normalization formula:

Normalized expression(NE)= Actual miRNA count/Total count of clean reads *1,000,000

After normalizing the expression, if the normalized expression value of a certain miRNA was zero, we change the value to 0.01. If the value was less than 1, we would not consider this miRNA in the further differential expression analysis. The values of fold-change and p-values, which were based on normalized expressions was used to draw the log2-ratio.

Fold-change formula:

Fold-change=log2(Young-NE/adult-NE)

P-value formula:

$$p(x|y)={\left(\frac{N_{2}}{N_{1}}\right)}^{y}{\frac{(x+y)!}{x!y!{\left(1+\frac{N_{2}}{N_{1}}\right)}^{\left(x+y+1\right)}}}_{D(y\leq y_{\text{max}}|x)\;=\;\overset{\infty }{\underset{y\geq y_{\text{max}}}{\sum }}p(y|x)}^{C(y\leq y_{\text{min}}|x)\;=\;\overset{y\leq y_{\text{min}}}{\underset{y\;=\;0}{\sum }}p(y|x)}$$

The N_1_ and x belong to the same sample. N_1_ represents the total count of clean reads and x represents the normalized expression level. The N_2_ and y represents the sample being compared to N_1_. N_2_ represents the total count of clean reads and y represents the normalized expression level [25].

### Predicted Target Genes

We predicted miRNA targets based on 3’UTR. Because the 3’UTR of the giant panda is not yet available, the 3’UTR of humans and dogs (*Canis familiaris*) were mapped to the giant panda genome with no more than 2 mismatches. We found that 80% length of the 3’UTR can be matched to the giant panda genome. If the site of the 3’UTR was near the 3’end of the giant panda gene, we considered that the 3’UTR was the 3’UTR of the giant panda. Additionally, we found the 3’UTR through Unigene Sequences of the giant panda [26]. We put these 3’UTR together to predict the miRNA targets using miRanda software [27].

### Analysis of KEGG and check the expression mRNA related to some pathways

We used the KEGG Automatic Annotation Server (KAAS) software [28] and KEGG mapper software [29] to annotate miRNA target genes. Subsequently, we used HISAT software [30] and StringTie software [31] to calculated the expression of the miRNA target genes and checked the expression of mRNAs whether is related to some special pathways. These mRNAs came from NCBI, the project Accession number is no.SRP041998 [26].

## Results

### Overview the characteristic of the small RNAs

**Fig 1:**
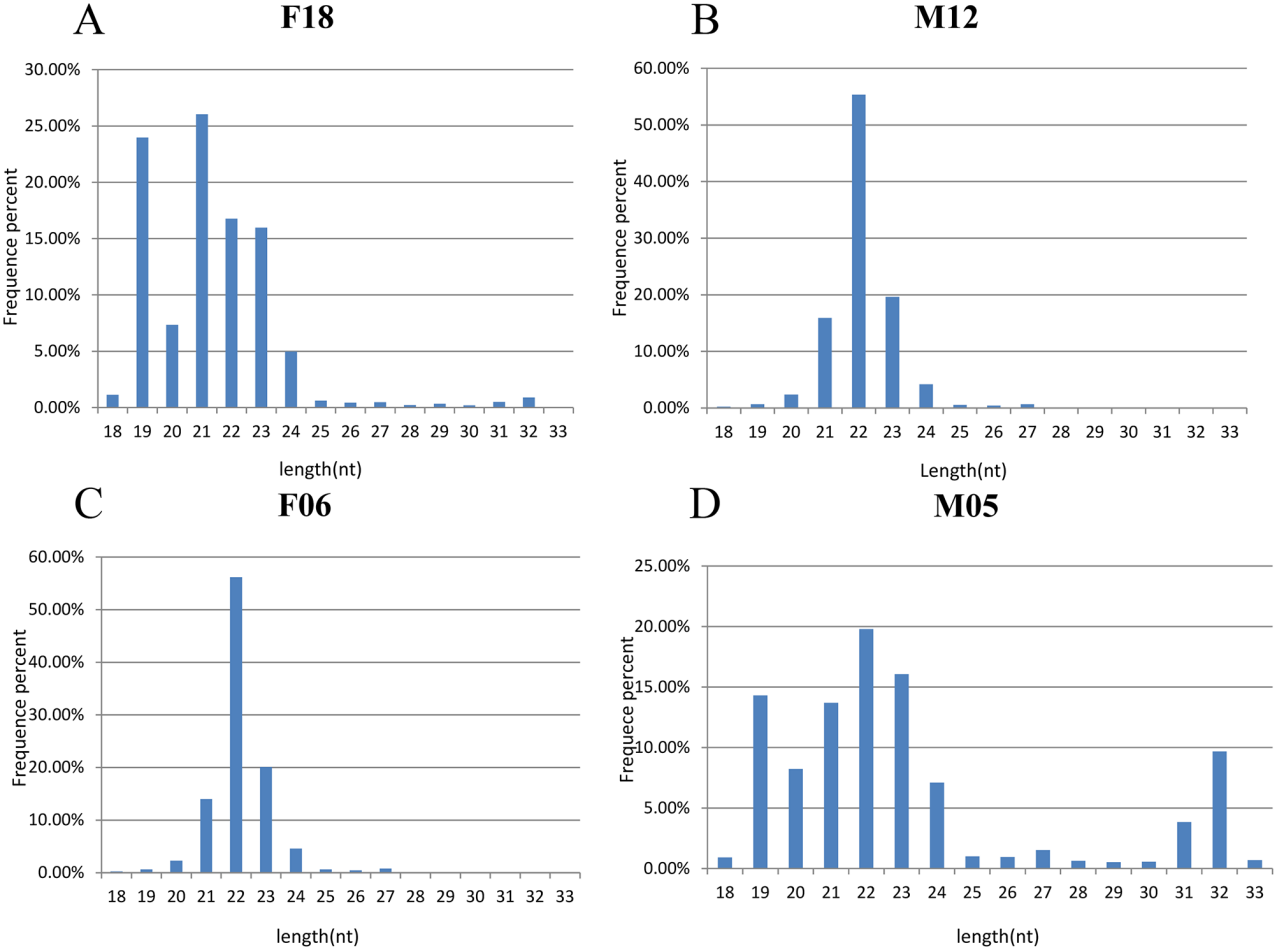
Frequency distribution of sequence lengths of sequencing results in all samples. (A) The lengths for F18. (B)The lengths for M12. (C) The lengths for F06. (D) The lengths for M05.

In order to characterize giant panda blood miRNA, the small RNAs (sRNA) from the blood of the four giant panda sampled (two young individuals and two adult individuals) were sequenced by Illumina NGS technology (Table 1). After filtering out low-quality sequences, a total of eleven million clean reads for F18 and seven million clean reads for M12 were retained in adult indivduals. Thirteen million clean reads for M05 and seven million clean reads for F06 were retained in young individuals (Table 2). The length distribution of the total clean reads in the all samples is shown in Fig 1. The majority of clean reads were 19-24nt in length, and the most abundant length size class was 22nt and 21nt. The number of 21nt miRNAs accounted for 26.04% in F18; The number of 22nt miRNAs accounted for 55.30% in M12; The number of 22nt accounted for 56.17% in F06; The number of 22nt accounted for 19.76% in M05. Subsequently, a total of 724,459 unique reads (39,325,353 total reads) were found in all samples (Table 2). Comparison of the total reads and unique reads revealed that 85% of the total reads were common in all samples and the left specific reads significantly less than the common total reads. Moreover, there are only about 1.5% unique reads were in all sample and most of the unique reads were sample-specifics.

**Table 2:** Summary of miRNAs sequencing.

	total number	unique number
**M05**	13570288	339479
**F06**	7093374	71211
**M12**	7101162	69609
**F18**	11560529	244160
**Total**	39,325,353	724,459

**Fig 2:**
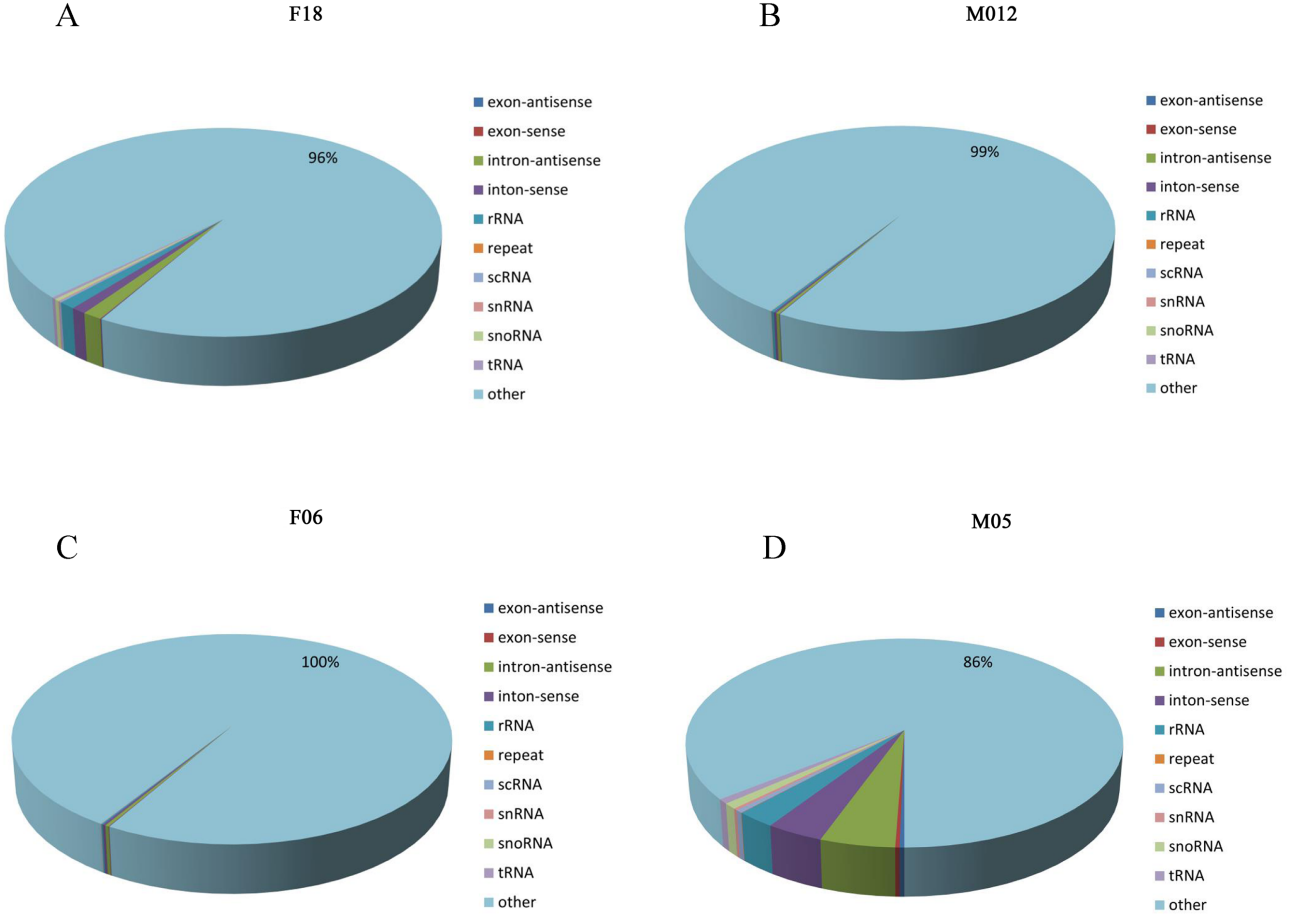
Category annotation for all samples. (A) Unique reads in F18. (B) Unique reads in M12. (C) Unique reads in F06. (D) Unique reads in M05.

After annotation of the sRNAs and removing the non-miRNA reads (rRNA, tRNA, snRNA, snoRNA, repeat sequence, exon and intron), there was a total of 339,479 unique reads in M05; 71,211 unique reads in F06; 69,609 unique reads in M12; 244,160 unique reads in F18. These require further analysis to identify miRNA candidates. The non-miRNAs accounted for 4% for F18, 1% for M12, 0% for F06 and 14% for M05 of unique reads (Fig 2).

### Identification of conserved and novel microRNAs

**Fig 3:**
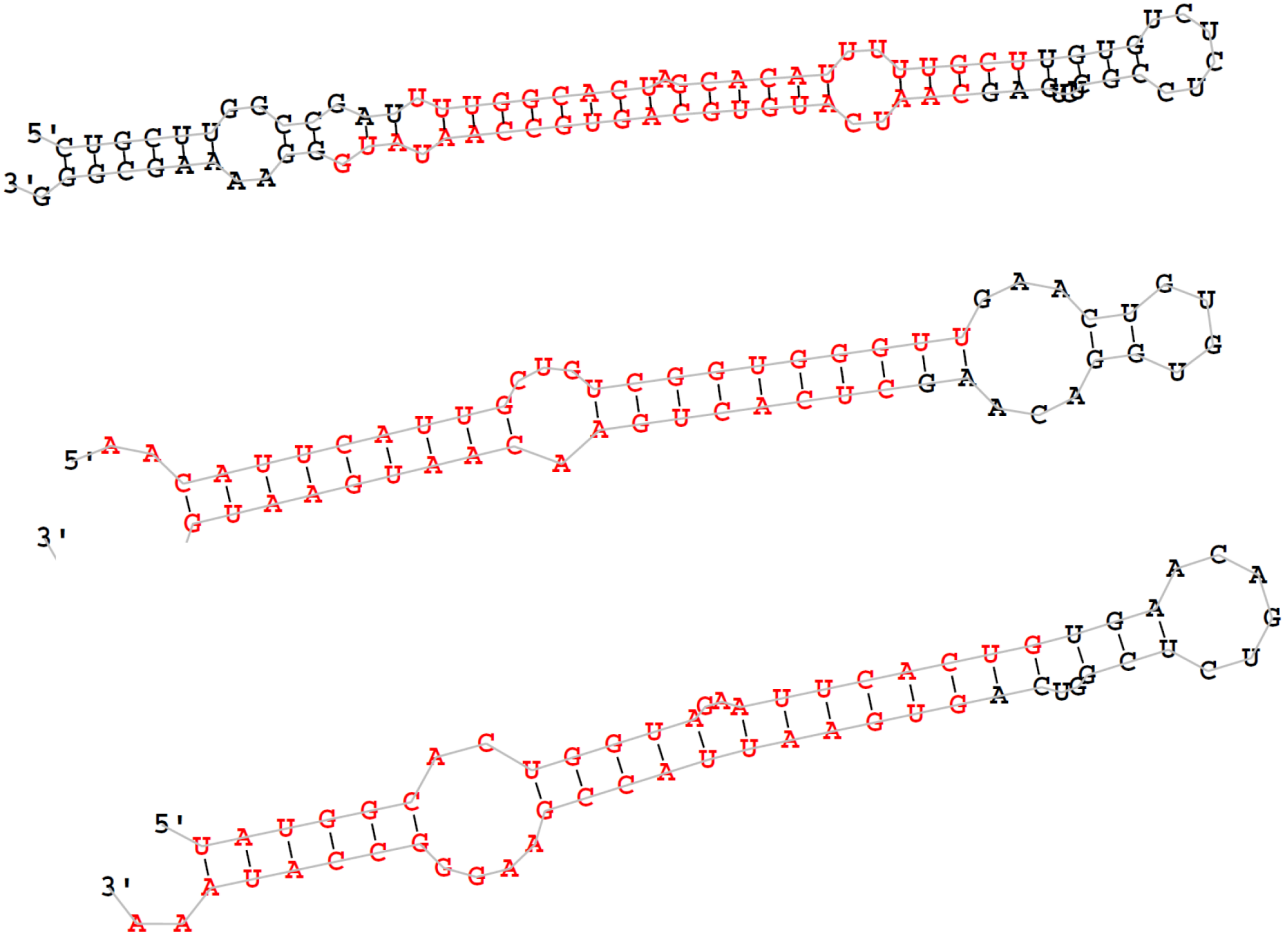
Partial secondary structure of novel miRNAs. The entire sequence represents pre-miRNAs.

To identify conserved miRNAs in these four giant panda blood samples, the unique reads which are miRNA candidates were compared to the conserved mammalian miRNAs (miRNA precursors and mature miRNAs) in the miRBase 21 database using miRDeep2 software. A total of 276 conserved sequences and 51 novel sequences were detected in four samples (S1 Table) (S2 Table). The 51 novel miRNAs have no homologous miRNAs in other mammals, and thus may be specific to the giant panda or have not identified yet. The typical secondary structures of 51 novel miRNA precursors were predicted by miRDeep2 software and shown in Fig 3.

### Expression characteristics of miRNAs in the young and adult giant panda blood

**Fig 4:**
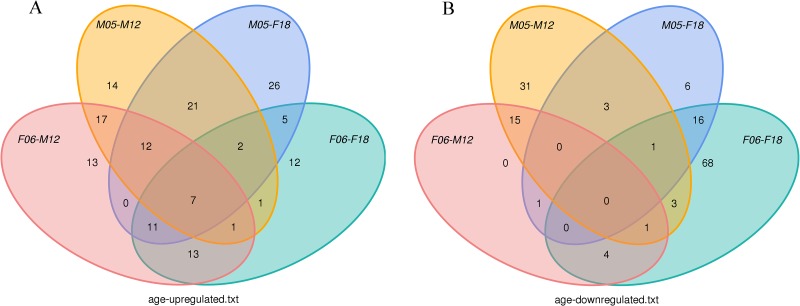
Differentially expressed miRNAs in the adult group and young individuals.

**Fig 5:**
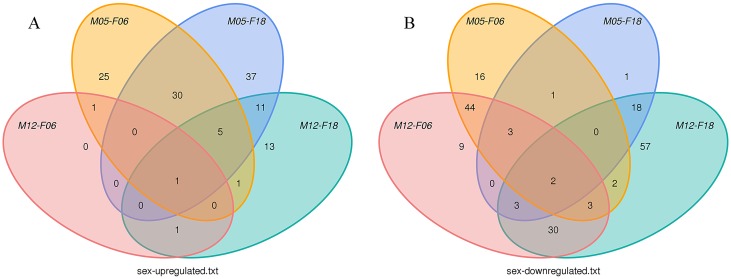
Differentially expressed miRNAs in the male group and female individuals.

As shown in Fig 4 and S3 Table, we noticed that the young individuals may have more up-regulated miRNAs than the adult: 74 miRNAs were up-regulated and 21 miRNAs were down-regulated in F06 compared to M12; 84 miRNAs were up-regulated and 27 miRNAs were down-regulated in M05 compared to F18; 75 miRNAs were up-regulated and 53 miRNAs were down-regulated in M05 compared to M12. Finally, we found that the expression levels of 7 miRNAs, including miR-192, miR-139 miR-664a, miR-331 and miR-301a, miR-129 and miR-24, were up-regulated in the young individuals. However, no miRNA with high expression level was observed in the pool of differentially expressed sequences. Moreover, we also had checked the expression difference of blood miRNAs between male and female giant pandas. We noticed that miR-29b was up-regulated in males compared to females, and miR-500 and miR-224 miRNAs were down-regulated in males (Fig 5). Therefore, the regulatory functions of miR-29b, miR-500 and miR-224 may be related to sex difference.

### Target prediction and pathway analysis of miRNAs in giant panda blood

A total of 4,602 target genes were predicted on the basis of all the miRNAs (S4 Table), among of which, 1,180 were regulated by 7 up-regulated miRNAs in the young group; 182 target genes were regulated by miR-29b and 417 target genes were regulated by miR-224 and miR-500. Some of these target genes were found to play an important role in immunity (S4 Table). For example, Eukaryotic Translation Initiation Factor 3 (*EIF3L*) was regulated by miR-664a and miR-301a. TGF-Beta Activated Kinase 1/MAP3K7 Binding Protein 1 (*TAB1*) were regulated by miR-664a and miR-331. *MAPK8* was regulated by miR-29b. Annexin A5 (*ANXA5*) was regulated by miR-192.

**Fig 6:**
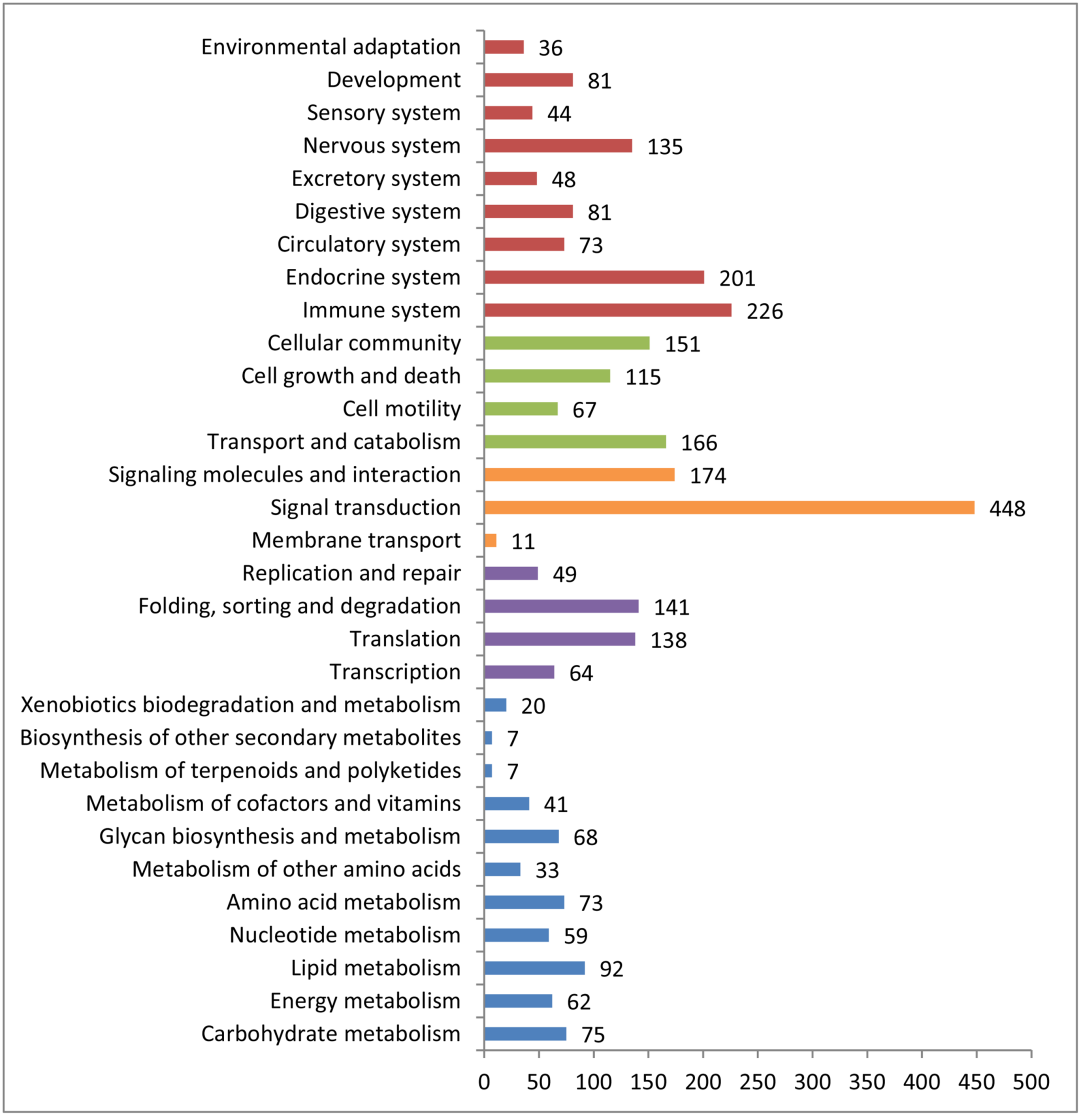
Pathway assignment based on KEGG for the giant panda target genes or miRNAs.

The predicted targets for all miRNAs and significantly differentially expressed miRNAs were annotated according to KEGG (Fig 6). Environmental information processing was the second most targets involved in. In environmental information processing, the three pathways of most targets involved were the Ras signaling pathway (76 targets genes of all miRNAs), the mitogen-activated protein kinase (MAPK) signaling pathway (84 targets genes of all miRNAs) and the phosphoinositide 3 kinase-Akt (PI3K-Akt) signaling pathway (101 targets genes of all miRNAs) (S5 Table).

### Expression of candidate target genes which were associated to significantly differentially expressed miRNAs

In most cases, miRNAs bind to the 3’UTR of target genes to inhibit it translation or inducing cleavage and degradation of target genes. In this study, after checking the relationship between the specific miRNAs and their target genes, we found that the expressions of 52 predicted target genes had higher trend in the young group, and 51 target genes with increased expressions were observed in the adult group; No differential expressed gene was found in the 182 candicate target genes of miR-29b; The expression levels of 34 predicted targets genes were highly expressed in the male giant pandas, and the expressions of 10 targets genes were highly expressed in female giant pandas, and it seems that all of these genes were simultaneously regulated by miR-224 and miR-500(S6 Table).

## Discussion

Although giant panda is one of the most beloved mammals in the world and has been the subject of many studies, as we known, no published study about giant panda miRNAs and their contribution to the immune system and diseases [32,33]. We sequenced the small RNAs in the blood of two young and two adult giant pandas using Illumina technology. We found that most of the predicted miRNAs are involved in immunity and diseases. For example, *EIF3L* was regulated by miR-664a and miR-301a, and it was a part of Eukaryotic initiation factor-3 (eIF-3) which played a key function in the initiation phase of protein synthesis [34]. On the other hand, *EIF3L* was a cellular factor stimulated by type I interferon response against invading viral pathogens [35]. Moreover, the expression of *TAB1* was predicted as under the regulation of miR-664a and miR-331, and this gene might be involved in the induction and maintenance of T-cell anergy trough mediating p38α activation [36]. Enforced expression of *TAB1* in a T-cell hybridoma resulted in decreased IL-2 and increased IL-10 production in response to activating stimuli, and it was consistent with an anergic phenotype [36]. At the same time, *MAPK8* was regulated by miR-29b. This target gene might be helpful in cancer therapy, because MAPK8 played a critical role in blunts apoptosis in cancer cells through autophagy [37]. In this research, seven miRNAs (miR-192, miR-139 miR-664a, miR-331 and miR-301a, miR-129 and miR-24) were significantly differentially expressed in the two groups and some of their targets were related to host immunity. Because the significantly differentially expressed miRNAs were associated with immunity and the expressions of most blood miRNAs with age were up-regulated in young and female individuals, this might mean young and female giant pandas appeared to show a stronger power to maintain the homeostasis.

The expressions of most blood miRNAs with age were up-regulated in young group (Fig 4). This result was comparable to the expression of *Caenorhabditis elegans* miRNAs, which decrease during cellular senescence [38]. Given this similarity, several studies had also shown this expression pattern in the serum of humans and rhesus monkeys (*Macaca mulatta*) with different ages [39]. This implies that miRNAs might serve as important regulators of age depending on expression levels of miRNAs. However, two researches indicated that 31 miRNAs were significantly down-regulated and 17 were up-regulated in the young mouse brain (*Mus musculus*) compared to the adult samples [2, 40]. A similar phenomenon showing that the expressions of miRNAs increase with age was also found in mouse liver (*Mus musculus*) [41, 42], which might be associated with tissue specific cellular senescence. This suggested that the regulations of miRNAs were very complicated and had organ-specific, namely, different organizations had different ways to be regulated. In blood, young individual’s immunity was typically strong and that the correlative miRNAs had a high expression so that some proteins associated with disease produce were produced less. For example, miR-664a, which can inhibit cancer cell migration and invasion [43], was significant difference in up-regulated miRNAs in young group compared to the adult group.

In addition to the effect on immune, we also predicted the targets of conserved and novel miRNAs. Signal transduction was the most targets involved in. In environmental information processing, Signal transduction was the most targets involved in. In environmental information processing, most target involved in pathways were the Ras signaling pathway, the MAPK signaling pathway, and the PI3K-Akt signaling pathway. All the three pathways have been found to be related to cancers [44]. Any aberrance in the Ras signal transduction pathway has been shown to be likely to contribute to human breast cancer [45]. Research also showed that inhibition of the MAPK signal pathway and the PI3K-akt pathway may lead to thyroid cancer cell apoptosis and to the arrest and apoptosis of pancreatic cancer cell cycles [46, 47]. The young giant pandas showed more targets of these three signaling pathways than old giant pandas. This might mean young giant pandas appear to show a stronger immunity to cancer than older giant pandas.

In addition, we identified 51 novel giant panda miRNAs. We found the target of these novel miRNAs were mainly involved in signal transduction. It was not surprising because the tendency was same with the conserved miRNAs. However, it was interesting that so many novel miRNAs were predicted that might be specific to the giant panda. The giant panda is considered to be the most primitive of living mammals and has some unique characteristics, such as its diet consists that is almost exclusively of bamboo [17], and thus may have more specific miRNAs. However, the relationship between miRNAs and characteristics of the giant panda remains a challenge for future research.

## Conclusions

In conclusion, in this study, we found a total of 327 miRNAs in four giant panda blood. Among them, 276 were conserved miRNAs and 51 were novel miRNAs. The targets of significantly differential expression miRNAs were mainly involved in the the PI3K-Akt signaling pathway, the MAPK signaling pathway, and the Ras signaling pathway of environmental information process. All three pathways (PI3K-Akt, MAPK and Ras) are related to cancer. The results underline the importance of miRNA expression in disease produce of giant panda, and raise the possibility that miRNAs can be used in some therapeutics for giant pandas. The novel miRNAs provide new steps for further study.

## Availability

Illumina sequencing data have been submitted to the Short Read Archive at NCBI and are accessible through accession no. SRP059993.

## Supporting Information

S1 Table Conserved miRNAs and their expression level which came from four samples. (XLSX)

S2 Table The novel miRNAs were predicted by miRDeep2 for four samples. (XLSX)

S3 Table Significantly differential expression miRNAs including sex and age. (XLSX)

S4 Table Predicted the targets of all miRNAs. (XLSX)

S5 Table The targets were involved in the three main pathways. (XLSX)

S6 Table The FPKM value of transcriptomes sequence which were same with miRNAs samples. (XLSX)
